# Antimicrobial Resistance Profiling of Biofilm Forming Non Typhoidal *Salmonella enterica* Isolates from Poultry and Its Associated Food Products from Pakistan

**DOI:** 10.3390/antibiotics10070785

**Published:** 2021-06-28

**Authors:** Abubakar Siddique, Sara Azim, Amjad Ali, Saadia Andleeb, Aitezaz Ahsan, Muhammad Imran, Abdur Rahman

**Affiliations:** 1 Atta Ur Rahman School of Applied Biosciences (ASAB), National University of Sciences and Technology (NUST), H-12, Islamabad 44000, Pakistan; mabubakar.asab@asab.nust.edu.pk (A.S.); saraazim94@gmail.com (S.A.); amjad.ali@asab.nust.edu.pk (A.A.); saadia.andleeb@asab.nust.edu.pk (S.A.); 2 Animal Health Program, Animal Sciences Institute, National Agriculture Research Centre, Park Road, Islamabad 44000, Pakistan; draitzaz786@gmail.com; 3 Department of Biosciences, Faculty of Sciences, COMSATS University Islamabad, Park Road, Islamabad 44000, Pakistan; m.imran@comsats.edu.pk

**Keywords:** poultry, *Salmonella* *enterica*, NTS, eggs, antibiotic resistance, MAR index, Pakistan

## Abstract

Salmonellosis caused by non-typhoidal *Salmonella enterica* from poultry products is a major public health concern worldwide. This study aimed at estimating the pathogenicity and antimicrobial resistance in *S. enterica* isolates obtained from poultry birds and their food products from different areas of Pakistan. In total, 95/370 (25.67%) samples from poultry droppings, organs, eggs, and meat were positive for *Salmonella.* The isolates were further identified through multiplex PCR (mPCR) as *Salmonella* Typhimurium 14 (14.7%), *Salmonella* Enteritidis 12 (12.6%), and other *Salmonella* spp. 69 (72.6%). The phenotypic virulence properties of 95 *Salmonella* isolates exhibited swimming and/or swarming motility 95 (100%), DNA degrading activity 93 (97.8%), hemolytic activity 92 (96.8%), lipase activity 87 (91.6%), and protease activity 86 (90.5%). The *sopE* virulence gene known for conferring zoonotic potential was detected in *S*. Typhimurium (92.8%), *S*. Enteritidis (100%), and other *Salmonella* spp. (69.5%). The isolates were further tested against 23 antibiotics (from 10 different antimicrobial groups) and were found resistant against fifteen to twenty-one antibiotics. All isolates showed multiple drug resistance and were found to exhibit a high multiple antibiotic-resistant (MAR) index of 0.62 to 0.91. The strong biofilm formation at 37 °C reflected their potential adherence to intestinal surfaces. There was a significant correlation between antimicrobial resistance and the biofilm formation potential of isolates. The resistance determinant genes found among the isolated strains were *bla_TEM-1_* (59.3%)*, bla_OxA-1_* (18%)*, bla_PSE-1_* (9.5%)*, bla_CMY-2_* (43%), and *ampC* (8.3%). The detection of zoonotic potential MDR *Salmonella* in poultry and its associated food products carrying cephalosporin and quinolone resistance genes presents a major threat to the poultry industry and public health.

## 1. Introduction

Non-typhoidal *Salmonella* (NTS) is one of the most important zoonotic foodborne pathogens [1] globallyGharieb, Tartor. About 2600 serovars of *Salmonella enterica* have been reported, which can cause disease in both animals and humans [2,3]. Gastroenteritis is the most common *Salmonella* infection in humans, accounting for 94 million cases each year, where 80.3 million are related to foodborne illnesses [4,5]. Major *Salmonella* outbreaks are caused by consuming contaminated poultry food (meat and eggs). At the same time, poultry farm handlers are also at risk due to direct or indirect contact with poultry birds [6,7]. Biofilm formation is important for the spread of NTS because biofilm-forming bacteria are resistant to drugs, disinfectants, and mechanical stress, making these biofilms a safety risk for the food industry. A variety of virulence factors and biofilm formation potential play an important role in the pathogenesis of *Salmonella* infection [8]. The irrational use of antibiotics in animal husbandry results in ever-increasing antimicrobial resistance in pathogens, including *Salmonella enterica*. Multidrug-resistant (MDR) and extended drug-resistant (XDR) *Salmonella* causes a serious threat to humans via transmission through the food chain [9]. Therefore, it is mandatory to monitor the antibiotic resistance patterns of *Salmonella enterica* in the food chain. Currently, third- and fourth-generation cephalosporin and fluoroquinolones are widely used to treat salmonellosis in humans and animals. However, the emergence of bacterial resistance to these clinically important antibiotics needs to be monitored [10,11,12]. In the Enterobacteriaceae family, resistance to cephalosporins is mainly linked with the production of large spectrum beta-lactamases such as ESBL (extended-spectrum beta-lactamases) and AmpC beta-lactamase [13]. Quinolone resistance is mainly associated with the mutations in quinolone resistance determining regions (QRDR) of *gyrA*, *gyrB*, *parC*, and *parE*. Extended-spectrum cephalosporin and fluoroquinolone-resistant Salmonella serovars have been isolated from food-producing animals and their products in many countries [14].

In Pakistan, there are very few reports regarding the pathogenicity and antimicrobial susceptibility pattern of *Salmonella* serovars from poultry farms. In addition, such studies were have been limited to one geographical region and targeted limited serovars for antimicrobial resistance. Therefore, it necessitates the surveillance of antibiotic resistance and biofilm potential and virulent characteristics of *S. enterica* serovars from poultry gut and poultry food from major cities across Pakistan. This study is the first report to the best of our knowledge which is based on samples from four major regions (Punjab, Sindh, Khyber Pakhtunkhwa (KPK), and Islamabad (Capital Territory) of Pakistan, where extensive commercial poultry farming is practiced. This study aimed to investigate the incidence, molecular detection of *Salmonella enterica* serovars, antibiotic resistance pattern, virulence factors, and biofilm potential of *Salmonella* isolated from poultry droppings, organs, and poultry products from commercial poultry farms and retail markets.

## 2. Material and Methods

### 2.1. Sample Collection

*Salmonella enterica* isolates were isolated from poultry droppings and poultry products, as previously described [14]. A total of 370 samples were collected between 2017 and 2018 from different commercial poultry farms and retail markets from various cities in Punjab, Sindh, KPK, and Islamabad (Capital Territory) of Pakistan. The samples were collected from birds indicating *Salmonella* infection symptoms, as confirmed by the farm resident veterinarian. Among 370 samples, 180 were from fresh droppings, 70 from poultry organs (liver, spleen, intestine, and ovary), and 60 samples each from meat and eggs. Sterile swabs were used for sampling from eggs and fresh feces, while 10 g meat and organs were collected aseptically. Samples were stored at 4 °C and transported to the lab for subsequent isolation.

### 2.2. Isolation of Salmonella Isolates

Fecal and egg samples were washed with 0.5 mL phosphate buffer saline (PBS), and 0.1 mL of them was inoculated in selective enrichment broth Selenite F broth (HIMEDIA, IND) and incubated at 37 °C for 24 h. For meat and organ samples, 1 g of each sample was mixed with 5 mL PBS and homogenized using pestle and mortar, and 1 mL of homogenized sample was mixed with 9 mL Selenite F broth and incubated at 37 °C for 24 h. After selective enrichment was completed, a serial dilution of each sample was made up to 10^−8^. 100 μL of enriched samples were spread on *Salmonella Shigella* (SS) agar (Oxoid, UK) plates and incubated at 37 °C for 24 h. Two or three suspected *Salmonella* black colonies on agar plates were picked to obtain purified isolates by further streaking method. Biochemical tests including Triple Sugar Iron (TSI), Citrate utilization, Urease, Sulphate, Indole, and motility tests were performed for preliminary screening of *Salmonella enterica* identification [15]. Overnight grown bacterial cultures were streaked on Triple Sugar Iron agar (Oxoid, UK.) Simmons Citrate agar (Oxoid, UK), Urease agar (Oxoid, UK), and Sulphate, Indole Motility (SIM) agar (HIMEDIA, IND) and were incubated at 37 °C for 24 h for subsequent biochemical characterization.

### 2.3. Molecular Detection of Salmonella using Multiplex PCR

The identified isolates were cultivated in Luria broth LB (Merck, Germany) and incubated at 37 °C for 24 h. Bacterial DNA was extracted using a DNA extraction kit (GF-1 Bacterial DNA Extraction Kit, Vivantis, Malaysia) according to the manufacturer’s instructions. For serovar identification, a multiplex PCR was performed. ST11–ST15 primers were selected from a randomly cloned gene and were specific to *Salmonella enterica. Sef*167-*Sef*478 primers were chosen from the *sefA* gene and were specific to *S*. Enteritidis. *Fli*15-*Tym* primers were selected from the *fliC* gene and were specific to *S*. Typhimurium. All primers for these genes were purchased from (Eurofins Scientific, France) (Table 1). The PCR reaction was carried out for initial denaturing at 94 °C for 5 min, 35 cycles of 94 °C for 30 s, 56 °C for 1 min 30 s and 72 °C for 30 s, followed by a final extension at 72 °C for 10 min [16]. Amplified PCR products were separated by electrophoresis on 1.5% agarose gel (bio-WORLD, USA). The gel was visualized under UV light, and images were analyzed with the Bio-Rad Gel Doc 1000 imager system.

### 2.4. Antimicrobial Susceptibility Assay

The antimicrobial susceptibility of *Salmonella* isolates was performed according to the Kirby-Bauer Disk Diffusion method as previously described [3]. Twenty-three antibiotics were selected based on clinical relevance, veterinary and poultry farm practices, which belong to different antimicrobial groups. The antibiotic discs (Oxoid, UK) used were as follows; amikacin (30 μg), chloramphenicol (30 μg), tetracycline (30 μg), cefixime (5 μg), amoxicillin/clavulanic acid (10 μg), ciprofloxacin (10 μg), gentamicin (30 μg), nalidixic acid (30 μg), cefepime (30 μg), trimethoprim/sulfamethoxazole (25 μg), ampicillin (30 μg), imipenem (10 μg), meropenem (10 μg), vancomycin (30 μg), streptomycin (25 μg), erythromycin (30 μg), linezolid (30 μg), rifampicin (30 μg), enrofloxacin (30 μg), oxacillin (5 μg), clindamycin (10 μg), minocycline (30 μg), and kanamycin (30 μg). *Salmonella* isolates were grown on Luria broth (LB) (Oxoid, UK) at 37 °C for 18 h. 100 μL of each overnight grown bacterial isolate was spread on 6-inch Muller–Hinton agar (MH) (Oxoid, UK) plates, antibiotic discs were placed on the agar plates and incubated at 37 °C for 18 h. Zones of inhibition were measured and interpreted by comparing with the breakpoints established for each antimicrobial according to the guidelines by the Clinical and Laboratory Standards Institute (CLSI 2017). Any isolate which has acquired non-susceptibility to at least one agent in three or more antimicrobial categories is considered multi-drug-resistant (MDR). The multiple antibiotic resistance index (MAR) was calculated as:

MAR index = No. of antibiotics resistant/No. of antibiotics tested

### 2.5. Molecular Detection of Antibiotic Resistance and Virulence Genes

Major antibiotic resistance genes in *Salmonella* isolates were identified using multiplex PCR. Different groups of antibiotics were selected, including beta-lactam, cephalosporins, and carbapenems (*bla CMY-2, blaOXA-1, bla PSE-1, bla TEM-1, bla NDM-1*, and *ampC*), and the zoonotic potential virulence gene *sopE* was targeted. The primer sequences and annealing temperature conditions were showed (Table 2). PCR conditions (except annealing temperature) of all target genes were: initial denaturation at 94 °C for 5 min followed by 30 cycles of denaturation at 94 °C for 30 s, primer annealing at a specific temperature for 45 s and extension at 72 °C for 30 s. The final extension step was done at 72 °C for 8 min [17].

### 2.6. Biofilm Characterization of Salmonella Isolates

The biofilm-forming potential of *Salmonella* isolates was determined by using a 96 well microtiter plate method as described previously [22], with slight modifications. Two-hundred microliters of Tryptic Soy Broth (TSB) and 20 μL overnight grown *Salmonella* culture was poured into 96 well microtiter plate. Plates were incubated at 30 °C and 37 °C separately for 48 h. Each well was washed twice with sterile phosphate-buffered saline (PBS) to remove planktonic cells. The remaining cells were fixed with 200 μL of methanol for 15 min. Wells were allowed to air dry and stained with 200 μL of 2% crystal violet for 30 min. The wells were carefully washed with distilled water to remove the excess stain. Plates were allowed to dry at room temperature. Dye bound to adherent cells were solubilized with 150 μL of 30% acetic acid. 30% acetic acid was taken as a negative control. A microplate reader (Bio-rad, USA) was used to read the plates at 620 nm wavelength. Three standard deviations above the mean OD of the negative control for the microtiter plate test were defined as the cut-off optical density (ODc). Isolates were classified as follows: (4 × OD_C_) < OD = strongly adherent, (2 × OD_C_) < OD ≤ (4 × OD_C_) = moderately adherent, OD_C_ < OD ≤ (2 × OD_C_) = weakly adherent, and OD ≤ OD_C_ = non-adherent [23].

### 2.7. Phenotypic Characterization of Extracellular Virulence Factors in Salmonella Isolates

*Salmonella* isolates were grown in tryptic soy broth (Oxoid, UK) and incubated at 37 °C for 24 h. For hemolytic activity, 100 uL, 0.5 McFarland bacterial suspensions were streaked on blood agar plates supplemented with 7% sheep blood and incubated for 24–48 h at 37 °C. Plates were observed for the formation of any clean (α-hemolysis) or greenish (β-hemolysis) hemolytic zones or no zone (γ-hemolysis). For lipase activity, 100 µL bacterial culture was streaked on tryptic soy agar supplemented with tween 80 and incubated for 24–48 h at 37 °C. Clear halo zones around bacterial colonies were taken as positive. For protease activity, 100 µL inoculum was poured on TSA plates supplemented with 1% casein from bovine milk (Sigma Aldrich, Germany) and incubated for 24–48 h at 37 °C. A clear zone because of casein hydrolysis was considered a positive result. For DNA degrading activity, 100 µL bacterial suspension was inoculated on DNase agar (Oxoid, UK). Plates were incubated for 24–48 h at 37 °C. The clear zone around colonies was considered positive for DNase activity [24].

### 2.8. Statistical Analysis

Spearman’s correlations between the number of MDR isolates and their biofilm formation were analyzed using SPSS version 20.0 software (IBM Corporation, Armonk, NY, USA). 

## 3. Results

### 3.1. Prevalence and Isolation of Salmonella Enterica Serovars

Among 370 samples, 26.7% (48/180) from poultry feces, 24.3% (17/70) from poultry organs, and 25% (30/120) from poultry meat and eggs were positive for *Salmonella* (Table 3). Colorless colonies with black center on SS agar plates were observed. The biochemical reactions on TSI agar slants were typical of *Salmonella* (alkaline slant and acidic butt and produce H_2_S). All 95 isolates were citrate and sulfate positive and negative for urease and indole tests, respectively. All *Salmonella* isolates exhibited swimming and swarming motility. Multiplex PCR of 95 isolates differentiated them into various *Salmonella enterica* serovars: *Salmonella* Enteritidis (12/95) 12.6%, *Salmonella* Typhimurium (14/95) 14.7%, and other *Salmonella* spp. (69/95) 72.6% (Table 3).

### 3.2. Antimicrobial Susceptibility Assay

**Figure 1:**
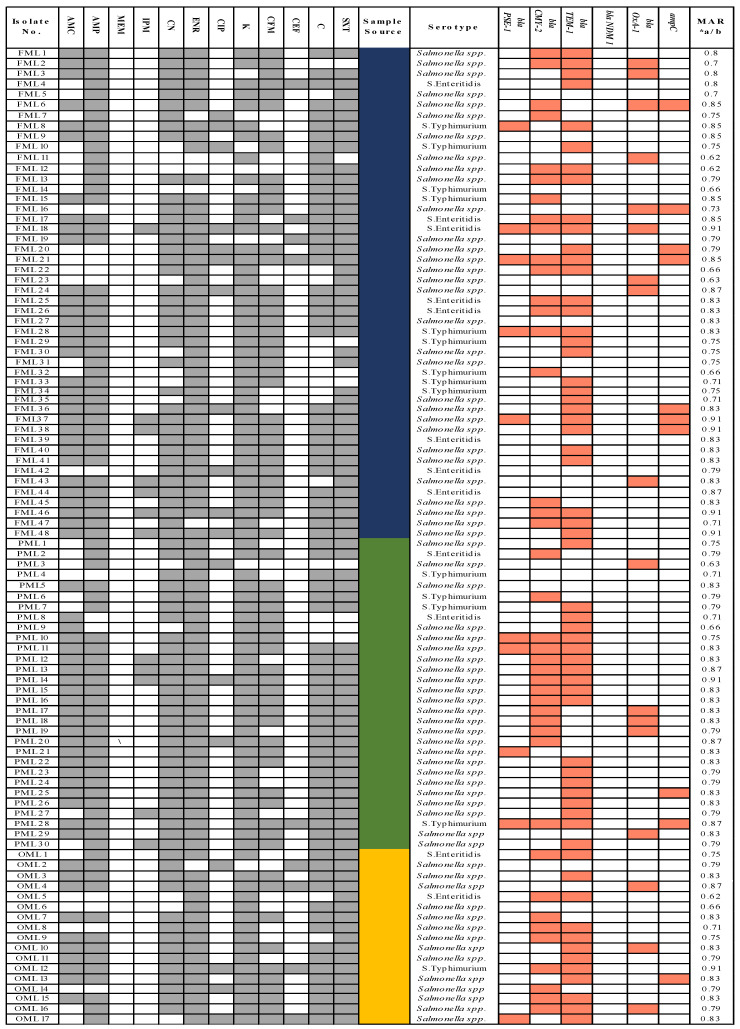
*Salmonella* strains (95) showing phenotypic antibiotic resistance profiles of 12 antibiotics, their source, origin, serotypes, and MAR index. Black squares indicate resistance; white squares indicate susceptibility; red squares indicate the presence of AMR genes; blue color presents isolates from poultry droppings, green; poultry food products (meat and eggs), yellow; poultry organs. Abbreviations: C: chloramphenicol (30 μg); AMC: amoxicillin-clavulanic acid (10 μg): CIP; ciprofloxacin (10 μg); CN: gentamicin (10 μg); SXT: sulfamethoxazole/trimethoprim (25 μg); K: kanamycin(30 μg): AMP: ampicillin (30 μg); MEM: meropenem (10 μg); IPM: imipenem (10 μg); CEF: cefepime (30 μg); CFM: cefixime (5 μg); ENR: enrofloxacin (10 ug); MAR: multiple antibiotic resistance; a*: No. of antibiotics resistant; b*: No. of antibiotics tested.

A high incidence of MDR was observed in all *Salmonella* isolates. Antimicrobial resistance pattern and MAR index of 95 *Salmonella* isolates were presented (Figure 1). All isolates were resistant against 11 antibiotics (oxacillin, clindamycin, erythromycin, streptomycin, nalidixic acid, fusidic acid, linezolid, rifampicin, tetracycline, minocycline, and vancomycin). Resistance to other antimicrobials was as follows: enrofloxacin, 95%; gentamycin, 93%; kanamycin, 91%; sulphamethoxazole/trimethoprim, 91%; ampicillin, 86%; amoxicillin/clavulanic acid, 81%; chloramphenicol, 81%; cefixime, 76%; ciprofloxacin, 19%; imipenem, 12%; cefepime, 9%; meropenem, 2% (Figure 1). Isolates from associated poultry products (meat and eggs) were highly resistant to different antibiotics, which is a public health concern. A high MAR index (0.62–0.91) was observed in *Salmonella*. MAR index of different *Salmonella* serovars was as follows: *S*. Typhimurium ranged from 0.66 to 0.87, *S*. Enteritidis ranged from 0.71–0.91, and in non-typeable *Salmonella* spp., it ranged from 0.62 to 0.91.

### 3.3. Distribution of Antibiotic Resistance and Virulence Genes in Salmonella Isolates

The presence and absence of antibiotic resistance genes are presented in (Figure 1). The results showed that *S*. Enteritidis harbored resistance genes for cephalosporin and carbapenems resistance *bla CMY-2* 7/12 (58.3%), *bla TEM-1* 8/12 (66.6%), *bla NDM-1* 0/12 (0%), and *ampC* 0/12 (0%); penicillin resistance genes *bla PSE-1* 1/12 (8.3%) and *bla OXA-1* 1/12 (8.3%); and the virulence gene of zoonotic importance *sopE* 12/12 (100%). *S.* Typhimurium exhibited cephalosporins and carbapenems resistance genes *blaCMY-2* 6/14 (42.9%), *bla TEM-1*9/14 (64.3%) *bla NDM-1* 0/14 (0%), and *ampC* 1/14 (7.1%); penicillin resistance genes *bla PSE*-23/14(21.4%), *bla OXA-1* 0/14 (0%), and *sopE* 13/14 (92.8%). Other *Salmonella* spp. exhibited cephalosporins and carbapenems resistance genes *bla CMY-2* 28/69 (40.5%), *bla TEM-1*, 40/69 (57.9%), *bla NDM-1* 0/69 (0%), and *ampC* 7/69 (10.1%); penicillin resistance genes *bla PSE-1* 6/69 (8.6%), *bla OXA-1* 16/69 (23.1%), and *sopE* 48/69 (69.5%).

### 3.4. Biofilm Formation Potential of Salmonella Isolates

**Figure 2:**
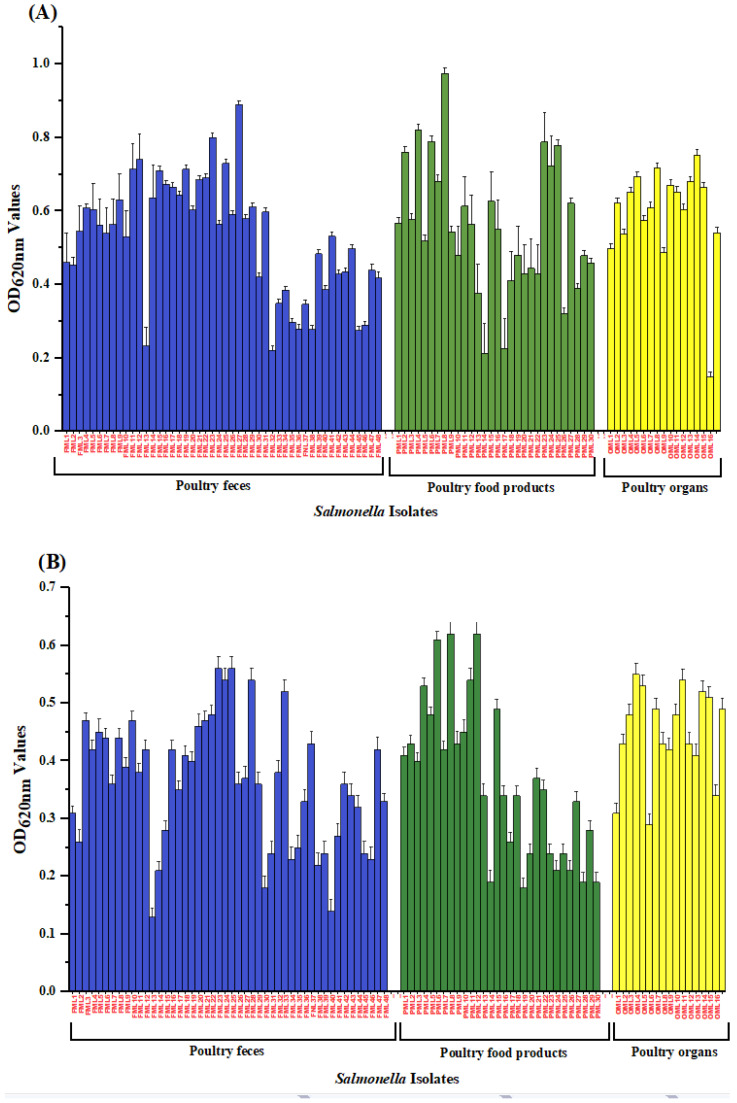
Results of biofilm formation assay. *X*-axis: *Salmonella* isolates from poultry droppings; poultry food products; poultry organs at two temperatures, (**A**): 37 °C; (**B**): 30 °C evaluated by crystal violet assay. Biofilm formation was assessed by staining the attached bacteria with 0.2% CV and measuring the OD values at 620 nm after 48 h growth. Error bars represent standard deviations between three replicates.

The Salmonella isolates’ biofilm formation was significantly influenced by the source of isolation, serotype, and incubation temperature. Biofilm formation of *Salmonella* isolates from different sources at two temperatures 30 °C and 37 °C is presented (Figure 2). The results revealed that poultry food isolates (meat and eggs) exhibited strong biofilm production at both temperatures. Biofilm potential was assessed at 30 °C and 37 °C for 48 h. Most of the *Salmonella* isolates of different origins showed strong biofilm at 37 °C for 48 h (Table 4). The data showed that the number of *S*. Typhimurium 11/14 (78.5%) with strong biofilm potential was almost double that of *S*. Enteritidis 5/12 (41.7%) at 37 °C for 48 h.

### 3.5. Correlation between the Number of Isolates Resistant to Antibiotics and the Ability to Produce Biofilms at Different Temperatures

To determine correlation between biofilm formation and the number of MDR *Salmonella*, Spearman’s rank correlation was used. All *Salmonella* isolates were biofilm producers at 30 °C, and they exhibited MDR profiles. The Spearman’s correlation coefficient (*r*_s_) in this case was 0.591 (*p <* 0.001). Similarly, all MDR *Salmonella* produced biofilms at 37 °C. The Spearman’s correlation coefficient (*r*_s_) comparison was 0.423 (*p <* 0.001). We found a significant correlation between biofilm formation and the number of multidrug-resistant isolates at 30 °C (*p* = 0.0001) and 37 °C (*p* = 0.00073).

### 3.6. Phenotypic Characterization of Extracellular Virulence Factors in Salmonella Isolates

Phenotypic characterization of external virulence factors of *Salmonella enterica* obtained from poultry feces and associated poultry products were shown (Table 5). All *Salmonella enterica* 95/95 (100%) in our study exhibited swarming and swimming motility. However, for *S.* Enteritidis isolates, 12/12 (100%) showed hemolytic activity, 11/12 (91.6%) displayed lipase activity, 12/12 (100%) showed DNA degrading activity, and 10/12 (83%) portrayed protease activity. For *S.* Typhimurium isolates, 13/14 (92.8%) had hemolytic activity, 11/14 (78.6%) showed lipase activity, 14/14 (100%) displayed DNA degrading activity, 10/14 (83%) portrayed protease activity. For non-typeable *Salmonella isolates* 67/69 (97.1%) exhibited hemolytic activity, 67/69 (97.1%) DNA degrading activity, 66/69 (95.6%) protease activity, and 65/69 (94.2%) had lipase activity.

## 4. Discussion

Non-typhoidal *Salmonella* gastrointestinal infections have become a major public health concern. Consumption of undercooked/semi-cooked poultry products is a major source of *Salmonella* infection in humans [25]. Poultry and poultry food products (meat and eggs) are a cheap source of high-quality protein for human consumption [26].

In our study, the overall incidence of *Salmonella* was 26% from poultry droppings, organs, and poultry food product samples, which emphasizes the monitoring of NTS at poultry farms as well as retail markets in Pakistan. The incidence of *Salmonella* in this study was higher than a previous study from Pakistan (12%), in other geographical regions, China (20%), Trinidad, Spain (20.5%), and Japan (7.9%) [27]. However, it was lower compared to those detected in India (33.1%), Canada (40%), Oklahoma (41%), Burkina Faso (55%), and Myanmar (97.8%) [20]. According to our study, we found non-typeable *Salmonella* spp. (73%), *Salmonella* Typhimurium (15%), and *Salmonella* Enteritidis (13%). However, these results are similar to another study in Faisalabad, Punjab, where *S.* Typhimurium and *S.* Enteritidis prevalence was found 28.4% and 9.2%, respectively [28]. A study from China revealed that *Salmonella* Enteritidis was the most abundant serovars, followed by *S.* Heidelberg and *S*. Typhimurium [19]. A similar study in Saudi Arabia showed the highest prevalence of *S*. Enteritidis (39.4%), followed by *S.* Paratyphi (21.2%), *S*. Typhimurium (15.2%), *S.* Typhi, and *S*. Arizona (12.1%), respectively. These *Salmonella* isolates were isolated from environmental and clinical samples. 

The emergence of antibiotic resistance in NTS is important for therapeutic control during the outbreak. 100% resistance against 11 antibiotics (oxacillin, clindamycin, erythromycin, streptomycin, nalidixic acid, fusidic acid, linezolid, rifampicin, tetracycline, minocycline, and vancomycin) is alarming. This may be the result of the irrational use of antibiotics in poultry farming and the healthcare system. A previous study found *Salmonella enterica* was intrinsically resistant only to oxacillin [29], and in another, *Salmonella* from chicken meat and giblets in Egypt were 100% resistant to erythromycin, penicillin, and amoxicillin. In comparison, 98.8%, 96.4%, 95.2%, and 91.6% were resistant to nalidixic acid, sulphamethoxazole, oxytetracycline, and ampicillin [30]. According to another study, the highest resistance was found against erythromycin (100%) and streptomycin (100%) [31]. In the present study, we report resistance against enrofloxacin, gentamycin, kanamycin, sulphamethoxazole, trimethoprim, ampicillin, amoxicillin/clavulanic acid, chloramphenicol, and cefixime in the range of 76% to 96%. This resistance pattern was similar to one of the previous studies where antimicrobial resistance to amoxicillin/clavulanic acid (96%), kanamycin (88%), ampicillin (85%), and cephalothin (81%) was observed [32]. In this study, we found phenotypic resistance against imipenem (12%), cefixime (76%), and cefepime (9%). The emergence of resistance to carbapenems, third- and fourth-generation cephalosporin in NTS has not been reported previously from this region. Carbapenems are considered as only beta-lactam antibiotics that are considered effective against MDR pathogens [33]. The increasing spread of carbapenems, third- and fourth-generation cephalosporin resistance in NTS may spread to typhoidal *Salmonella* and other nosocomial enteric pathogens because they inhabit the same environment [34]. According to a previous study, a fourth-generation cephalosporin and fluoroquinolones resistant *Salmonella typhi* was reported in the Sindh region, Pakistan [35]. Another study from the Sindh region also revealed quinolones and cephalosporins resistance in NTS isolates from poultry meat [36]. Further, WGS analysis may reveal the source of such resistance in different *Salmonella* serovars [37]. Detection of MDR *Salmonella* isolates in this study warrant more attention towards surveillance of antibiotics usage in agriculture and human health care sectors in Pakistan. In our study, a high MAR index detection could be attributed to the increased use of clinically important antibiotics for bacterial infection control in humans and as therapeutic agents or growth promoters in veterinary practice for livestock in Pakistan. [38]. The high MAR observed in a similar study from Brazil ranged from 0.18–0.40 in different *Salmonella* serovars isolated from poultry sources [39]. MAR index of *Salmonella* isolates from seafood ranged from 0.14–0.45 in a study from Malaysia [40]. MAR index ranged from 0.21–0.46 in different *Salmonella* serovars isolated from ready-to-eat shrimps from a study in Nigeria [41]. A high number of tested antibiotics and high antibiotic resistance detection in our study compared to previous studies may be the cause of the high MAR index. The rapid emergence of antibiotic resistance is attributed to the selective pressure of antibiotics through evolutionary responses due to natural selection [42].

The emergence of extended-spectrum β-lactam/cephalosporin resistance in *Salmonella* can narrow its control options by antibiotics. Most of the antibiotic-resistant determinants are present in plasmids or integrons, which can transfer these genes to other bacterial species of different or the same group [43]. In the present study, among cephalosporin and carbapenems resistance genes in *S*. Typhimurium, *bla TEM-1* (64.3%) is more abundant than other genes. In *S.* Enteritidis*, bla TEM-1* (66.6%) was detected as the most prevalent. This is related to another study where *bla TEM-1* (35.3% and 72.7%) was detected as most prevalent in *S.* Typhimurium and *S*. Enteritidis isolates, respectively [28]. There are various reports in previous studies where the *bla TEM-1* gene was detected as the most prevalent [44]. According to our study, the beta-lactamase penicillin gene *bla* PSE-1 gene (21.8%) was most prevalent in *S.* Typhimurium. While in *S.* Enteritidis, *bla PSE-1* (8.3%) and *bla OXA-1*(8.3%) were found in the same proportion. The mismatch between genotypic and phenotypic antibiotic resistance in our study may be due to mutation in genes and variation in gene expression within different *Salmonella* isolates. The *sopE* gene was found in all S. Enteritidis and 92.8% of *S*. Typhimurium, whereas in non-typeable *Salmonella*, it was observed in 68.1% isolates, which agrees with previous findings [45,46]. The *sopE* gene is encoded in SPI-1. It is identified in the isolates that are mainly involved in major epidemics; therefore, *sopE* has been identified as a major determinant in the spread of epidemic strains [47]. In another study, *Salmonella* Enteritidis isolated from chicken, eggs, and humans constitute the *sopE* gene, which may indicate its importance in zoonosis [48].

Biofilm formation may help in *Salmonella* survival in poultry farms and poultry food products [49]. In our study, *S*. Typhimurium (78.5%) exhibits strong biofilm potential at 37 °C; similar results were revealed from previous studies [19,50]. *Salmonella* isolated from poultry food (meat and eggs) in our study with moderate to strong biofilm potential and having MDR characteristics is a concern for public health and poultry farming. Bacteria grown in biofilms have a greater ability to transfer genes horizontally than planktonic cells [51]. Biofilms increase the chances of gene transfer with the help of virulence factors and antibiotic-resistant genes from resistant to susceptible bacterial species, which leads to the emergence of new antibiotic resistance in pathogens [21]. The variation in the biofilm potential of the *Salmonella* isolates in this study may be due to the difference in incubation temperature (30 °C and 37 °C) coupled with species diversity as described in a previous study [52]. The reason for choosing temperatures of 30 °C and 37 °C to assess biofilm formation potential was because sampling regions fall in the temperate zone, and summer temperatures are high. Most poultry meat and egg shops operate in warm climatic conditions where temperatures lie between 28 °C to 30 °C. This study found a significant correlation between antibiotic resistance and biofilm formation at both temperatures 30 °C and 37 °C. Such a relationship has been described for other bacteria, although the findings were sometimes inconsistent, and the correlations were species-dependent [53]. Our data showed that the *Salmonella* serovars from poultry gut, organs, and food (meat and egg) had virulence characteristics and determined the pathogenicity of *Salmonella* isolates. These external virulence factors are swimming and swarming motility, hemolysis, lipase, the presence of protease, and DNA degrading activity. Motility is an important pathogenic property of bacteria that is closely related to virulence factor production, antibiotic resistance, and biofilm potential [54]. According to a previous study, a significant linkage between protease production, motility, and pathogenesis has been reported [55]. Extracellular protease, DNA structure, and lipolytic activity positively correlated with biofilm formation [56,57,58].

Management strategies for antibiotic resistance should be adopted to control the dissemination of antibiotic resistance includes the following: improved information to enhance the awareness, control of non-therapeutic use of antibiotics in food animal production system, improvement in diagnostic procedures, and enhancement of microbiological laboratory equipment and personals [59,60]. These suggestions may assist in the reduction of antibiotic resistance and can improve the public health sector in Pakistan.

## 5. Conclusions

These findings indicated highly antibiotic-resistant NTS serovars with zoonotic potential at local poultry farms in Pakistan, emphasizing the need to adopt more biosecurity measures, environmental and personal hygiene awareness among the local poultry farmers. The detection of the most important foodborne zoonotic *Salmonella enterica* serovars Typhimurium and Enteritidis is of public health significance. The emergence of MDR *Salmonella* serovars is of great concern for the targeted antimicrobial therapy. Resistance from these *Salmonella* isolates may transfer to typhoidal *Salmonella* and other Enterobacteriaceae family as these pathogens share a common environment for propagation. Irrational usage of different antibiotics in the poultry industry should be checked to avoid spreading and disseminating antibiotic resistance. The biofilm formation potential of these isolates is of great concern for the food industry and public health.

## Figures and Tables

**Table 1:** Primers used for *Salmonella* detection with Multiplex PCR.

Target Sequence	Target Strain	Primer Sets	Length	Primer Sequence 5′----3′	Amplification Region	Reference
Random Sequence	*Salmonella* spp.	*ST11* *ST15*	24 24	GCCAACCATTGCTAAATTGGCGCA GGTAGAAATTCCCAGCGGGTACTGG	429	[17]
*fliC* gene	*Salmonella* Typhimurium	*Fli15* *Tym*	22 22	CGGTGTTGCCCAGGTTGGTAAT ACTCTTGCTGGCGGTGCGACTT	559	[17]
*sefA* gene	*Salmonella* Enteritidis	*Sef 167* *Sef 478*	20 20	AGGTTCAGGCAGCGGTTACT GGGACATTTAGCGTTTCTTG	312	[17]

**Table 2:** Primers and PCR conditions used for antibiotic resistance genes and virulence factor detection.

Genes	Sequences (5′-3′)	Annealing Temp. (°C)	Amplicon Size	References
*bla PSE-1*	CGCTTCCCGTTAACAAGTAC CTGGTTCATTTCAGATAGCG	50	430	[18]
*bla CMY-2*	TGGCCAGAACTGACAGGCAAA TTTCTCCTGAACGTGGCTGGC	57	870	
*bla TEM-1*	CAGCGGTAAGATCCT TGAGA ACTCGCCGTCGTGTAGATAA	55	643	[19]
*bla OxA-1*	ATGAAAAACACAATACATATC AATTTAGTGTGTTTAGAATGG	50	830	
*bla NDM-1*	GGG CAG TCG CTT CCA ACG GT GTA GTG CTC AGT GTC GGC AT	58	475	[20]
*ampC*	AACACACTGATTGCGTCTGAC CTGGGCCTCATCGTCAGTTA	60	1226	[9]
*SopE*	ACACACTTTCCACGAGGAAGCG GGATGCCTTCTGATGTTGACTGG	50	398	[21]

**Table 3:** Prevalence of *Salmonella* in poultry feces, poultry organs, and poultry food products.

Sr. No.	Sample Source	No. of Samples (*n*)	Positive Samples (%)	Serovar (s) Isolated	No. of Serovar out of Positive Sample (%)
1	Poultry feces	180	48 (26.7)	*S.*Typhimurium	9 (18.75)
				*S.* Enteritidis	8 16.6)
				Other *Salmonella*	31 (64.6)
2	Poultry organs	70	17 (24.3)	*S.*Typhimurium	1 (5.8)
				*S.* Enteritidis	2 (11.7)
				Other *Salmonella*	14 (82.3)
3	Poultry Meat	60	21 (35)	*S.*Typhimurium	2 (9.5)
				*S.* Enteritidis	1 (4.7)
				Other *Salmonella*	18 (85.7)
4	Poultry eggs	60	9 (15)	*S.*Typhimurium	2 (22.2)
				*S.*Enteritidis	1 (11.1)
				Other *Salmonella*	6 (66.6)

**Table 4:** Biofilm potential of different *Salmonella* isolates at different temperatures.

*Salmonella* spp.	Temperature (°C)	Weak Biofilm	Moderate Biofilm	Strong Biofilm	No Biofilm
*S.* Typhimurium (*n* = *14*)	30	3 (21.4)	4 (28.5)	7 (50)	0 (0)
	37	1 (8)	2 (14.2)	11 (78.5)	0 (0)
*S.* Enteritidis (*n* = *12*)	30	2 (16.6)	7 (58.3)	3 (25)	0 (0)
	37	2 (16.6)	5 (41.7)	5 (41.7)	0 (0)
Other *Salmonella* spp. (*n* = *69*)	30	18 (26.1)	30 (43.4)	21 (30.4)	0 (0)
	37	11 (15.9)	20 (28.9)	38 (55.1)	0 (0)

**Table 5:** Phenotypic virulence characteristics of *Salmonella* isolates.

*Salmonella* spp.	DNA Degrading Activity	Hemolytic Activity	Lipase Activity	Protease Activity	Swimming Motility	Swarming Motility
*S.* Typhimurium (*n* = 14)	14 (100)	13 (92.8)	11 (78.6)	10 (83)	14 (100)	14 (100)
*S.* Enteritidis (*n* = 12)	12 (100)	12 (100)	11 (91.6)	10 (83)	12 (100)	12 (100)
Other *Salmonella* spp. (*n* = 69)	67 (97.1)	68 (98.5)	65 (94.2)	68 (98.5)	69 (100)	69 (100)
